# Implementation of a Multidevice Telemonitoring Program for Home-Based Nursing Care in Quebec: Qualitative Report

**DOI:** 10.2196/83615

**Published:** 2026-06-16

**Authors:** Alexandre Castonguay, Guy Paré, Marie-Pierre Moreault, Marie-Soleil Hardy, Mickaël Ringeval, Philippe Voyer

**Affiliations:** 1 Faculty of Nursing, Université de Montréal, 2375, chemin de la Côte-Sainte-Catherine, Montréal, QC, H3T 1A8, Canada, 1 514-343-6437; 2 Research Chair in Digital Health, HEC Montréal, Montréal, QC, Canada; 3 HEC Montréal, Montréal, QC, Canada; 4 Faculty of Nursing, Université Laval, Québec, QC, Canada; 5 Computer Information Systems Department, Bentley University, Waltham, MA, United States

**Keywords:** telemonitoring, remote patient monitoring, home care services, implementation science, medical informatics applications, qualitative research, nursing practice.

## Abstract

**Background:**

Home care services are under increasing pressure due to population aging, rising chronic disease prevalence, and workforce shortages. Digital health technologies, particularly home telemonitoring systems, are seen as promising tools to detect early clinical deterioration, reduce hospital use, and support continuity of care. However, real-world evidence on the implementation of such technologies in public home care systems remains limited. In 2023‐2024, the Quebec Ministry of Health and Social Services funded a pilot to implement a multidevice telemonitoring intervention for older adults with heart failure across three integrated health and social services centers (CISSS). The initiative aimed to assess feasibility, acceptability, and the organizational conditions shaping implementation.

**Objective:**

This study documents the real-world implementation of a multidevice telemonitoring program from the perspective of home care nurses, with complementary insights from managers. It identifies perceived barriers, enablers, and contextual strategies for adaptation and sustainability.

**Methods:**

We conducted a qualitative study guided by the iCHECK-DH framework and principles of implementation science. Data sources included 30 semistructured interviews, 19 with home care nurses, and 11 with clinical and administrative managers. The intervention combined four connected devices (ie, Bluetooth scale, smartwatch, xPill adherence system, and voice-activated tablet), monitored by nurses through the Virtuose dashboard. Data were analyzed thematically, focusing on implementation processes, user experience, and organizational integration.

**Implementation (Results):**

The intervention involved 67 patients initially recruited (34 completed the 6-mo intervention) and was implemented across three home care organizations. Qualitative findings were based on 30 semistructured interviews with home care nurses (n=19) and administrative managers (n=11). About 16/21 (76%) of nurses accessed the dashboard daily or near-daily, but challenges included alert fatigue, workflow disruption, and limited integration with clinical systems. Nurses described mixed initial reactions to telemonitoring, valued hands-on training, and emphasized the need for clearer workflows and technical support. Digital tools were seen as clinically useful but added complexity to care delivery. Sustained use required local adaptation, attention to digital literacy, and greater system integration. Relational care remained central to nursing practice, even in digitally supported contexts.

**Conclusions:**

Telemonitoring programs can be successfully implemented in public home care settings when supported by strong leadership, responsive training, and adaptive workflows. This study highlights the relational, technical, and organizational dimensions of implementation and offers lessons to inform future scale-up efforts in similar public health systems.

## Introduction

As health systems face aging populations, workforce shortages, and rising chronic disease burdens, new models of care delivery are urgently needed. Heart failure (HF), specifically, is a chronic condition that poses major challenges for both patients and healthcare systems. Characterized by frequent exacerbations, high rates of emergency visits and hospitalizations, and substantial morbidity and mortality, HF demands intensive management [1].

Digital health technologies, including home-based telemonitoring, offer promising ways to support clinical follow-up and ease pressure on conventional infrastructure [2-5]. Studies have associated telemonitoring with improved quality of life [6], reduced depressive and anxiety symptoms [7,8], increased adherence to treatment regimens [7,9], and better disease knowledge and self-management [6,9-12]. These gains may contribute to lower HF-related mortality [6,7,13-16], fewer hospitalizations and emergency visits [13,17-19], and shorter hospital stays [17,20].

Yet, real-world adoption remains limited, and reviews have highlighted a lack of empirical studies examining how such digital health interventions are implemented in routine practice, particularly for chronic disease management [2,3].

Prior Quebec studies have evaluated home telemonitoring for chronic obstructive pulmonary disease [5] and hypertension [12], but to our knowledge, no implementation of multidevice telemonitoring for heart failure had previously been conducted within Quebec’s public home care system. The present pilot extends this body of work by combining four connected devices, a centralized nurse-facing dashboard, and integration into routine CISSS home-care workflows across three regions.

Furthermore, technology alone cannot meet the complex and evolving needs of patients receiving home care, especially those with progressive chronic illness. Nurses are critical to the success of digital health interventions, serving as the primary clinical contact in home settings. In telemonitoring models, their role extends beyond direct care to include data interpretation, alert triage, follow-up coordination, and patient education [21]. Their ability to integrate new digital tools into daily workflows, maintain continuity, and respond dynamically to system feedback makes them key mediators between innovation and practice. Understanding their experience is essential to evaluating the feasibility, sustainability, and safety of implementation.

This study examines the implementation of a multidevice telemonitoring program for older adults with heart failure across three regional health organizations in Quebec, Canada. The intervention combined four connected devices (Bluetooth scale, smartwatch, xPill adherence system, and voice-activated tablet) monitored via a centralized digital dashboard, as part of a pilot sponsored by the Quebec Ministry of Health and Social Services (MSSS).

While a companion paper [22] reports patient and caregiver experiences, the present article focuses on home care nurses and managers as the primary agents of implementation. This article reports on the barriers, enablers, and contextual adaptation strategies that shaped implementation from their perspective.

## Methods

### Study Aim and Design

This qualitative implementation study examined the deployment of a multidevice home telemonitoring system for older adults with heart failure across three regional health organizations in Quebec. It aimed to explore feasibility, acceptability, and integration into nursing practice, as well as barriers, enablers, and contextual adaptation strategies related to workflow, digital tools, and long-term sustainability, primarily from the perspective of home care nurses, with complementary input from managers. Although the broader initiative included quantitative and qualitative components, this article focuses on the lived experience of implementation and does not report formal key performance indicators.

### Study Context

This study was conducted as part of the pilot project, implemented across three CISSS in Quebec. Sites were selected by the Quebec MSSS to reflect diverse home care delivery contexts (eg, rural vs urban). A multisite governance structure coordinated implementation, which was staggered but unified by shared protocols, training materials, and technical platforms. The pilot was mandated by the *Direction générale des aînés et des proches aidants* of the Quebec MSSS to generate real-world evidence on multidevice home telemonitoring with a view to a potential province-wide scale-up; in iCHECK-DH terms, the implementation is at the *Piloting and Evidence Generation* stage.

The intervention supported older adults with heart failure through remote monitoring of physiological and symptomatic data, caregiver engagement, and clinical follow-up by 21 home care nurses.

Managers from the three CISSS were involved in project development and local implementation planning. Each site determined how telemonitoring would be incorporated into nursing practice: two required nurse participation, whereas one used a voluntary model. Managers also identified eligible patients, defined as French-speaking adults aged 65 years or older receiving home care for heart failure and without neurocognitive disorder. Patients and caregivers who agreed to participate were then contacted by a research professional, who provided study information and obtained consent.

Finally, a total of 67 patients were initially recruited for the intervention. Each morning, patients received an alert to complete a symptom questionnaire, which they could complete by touch or voice. They also weighed themselves daily using the Bluetooth scale. The xPill adherence system tracked medication intake via pharmacy connectivity.

### Intervention and Nurse Involvement

The telemonitoring solution relied on proprietary, closed-source platforms developed by Virtuose Technologies and DOmedic, which remained vendor-owned throughout the pilot. The two platforms were selected through a co-development partnership formalized at project inception—the founder of Virtuose Technologies was a project co-instigator alongside the academic lead—and equipment was acquired via direct contracts between each CISSS and the technology partners under the ministerial research mandate, without a public tender. At acquisition, the smartwatch was Health Canada-approved; the connected scale was not. The Virtuose dashboard operated independently from existing clinical information systems used in Quebec, including i-CLSC and SyMO, with no Fast Healthcare Interoperability Resources (FHIR)-based interoperability standard to support data exchange with other health information systems. Physiological data were transmitted via patients’ home networks to the Virtuose cloud; alerts were generated automatically based on preconfigured thresholds. Community pharmacists accessed xPill adherence data only through DOmedic’s proprietary interface. A privacy impact assessment and cybersecurity review were completed before deployment.

Nurses played a central role in operationalizing the telemonitoring model. They were responsible for:

- Identifying and recruiting eligible patients from their caseloads
- Installing and configuring devices in patients’ homes
- Training patients and caregivers
- Monitoring data via the Virtuose dashboard

Prior to deployment, nurses completed two training components: a self-paced clinical module on geriatric cardiac assessment (approximately 4 h across 10 videos), and a 2-hour hands-on session covering the digital devices and the Virtuose dashboard. Additional support was provided by trainers from Virtuose Technologies (dashboard) and DOmedic (xPill adherence system). Several nurses also acted as super-users or local champions during implementation.

### Participants and Data Collection

This study draws on two categories of participants: (1) *Home care nurses *(n=19): recruited from the three participating sites, all nurses participated in post-implementation semi-structured interviews focused on their experience with the telemonitoring intervention; and (2) *Managers and coordinators* (n=11): interviews were conducted post-implementation to capture organizational perspectives on deployment outcomes, perceived impact on care delivery, and lessons learned for future scale-up. The average duration of interviews varied by participant type: between 20 and 30 minutes for home care nurses, and 60 and 75 minutes for managers and coordinators. All interviews were audio-recorded with consent and transcribed verbatim.

### Data Analysis

A directed thematic content analysis was conducted on the qualitative data obtained from the semi-structured interviews [23]. Interview recordings were transcribed with the aid of Whisper (OpenAI, local deployment), then checked against the audio by the research team before analysis. An initial coding framework, aligned with the study’s primary objective, focused on identifying barriers, enablers, and contextual strategies. The analysis was guided by sensitizing concepts from implementation science (eg, acceptability, feasibility, integration, and sustainability) and guided by the iCHECK-DH reporting framework [24]. The coding process was iterative and included constant comparison across participants, with attention to ensuring that insights reflected perspectives across all participating sites. Given the near-complete inclusion of eligible participants, data saturation was deemed to have been achieved when no new themes emerged in the final interviews across participant groups. This determination was confirmed through iterative team discussions. Codes relating to barriers, enablers, and contextual adaptation strategies were iteratively compared across participant groups and sites, then grouped into higher-order implementation themes through team discussion. Illustrative examples of the coding-to-theme process are provided in Multimedia Appendix 1.

### Ethical Considerations

This study received ethics approval from the Research Ethics Board of the CISSS de Chaudière-Appalaches, the lead institution for ethics review (MP-23-2023-1037), on July 11, 2023. The two additional participating institutions approved the project through institutional agreements recognizing the lead site’s review. All participants provided written or verbal informed consent, including permission for audio recording, anonymized data use, and secure data storage. Transcripts were deidentified, and data were stored on encrypted servers accessible only to the research team. Participants were assured that their decision to participate would not affect their employment or professional standing. No financial compensation was provided to participants.

The study was conducted in accordance with applicable Quebec provincial data protection requirements: Act respecting health and social services information, CQLR c R-22.1 [25]; Act respecting Access to documents held by public bodies and the Protection of personal information, CQLR c A-2.1 [26]; and Act to modernize legislative provisions as regards the protection of personal information, SQ 2021, c 25) [27]. A privacy impact assessment was completed to authorize limited data access for technology partners, and all digital tools underwent cybersecurity testing by institutional information security teams. External access to patient information, such as by community pharmacists, was permitted only with explicit patient consent. These safeguards ensured participant autonomy, data security, and institutional accountability throughout the project lifecycle.

## Implementation (Results)

Across the three participating sites, 67 patients were recruited and 34 completed the 6-month intervention. Each nurse followed 1 to 5 patients remotely, so telemonitoring represented a small part of their overall caseload. Findings are organized into four themes summarized in Table 1. As implementation context, about 16/21 (76%) of nurses accessed the dashboard daily or near-daily, and no formal tracking of alert volumes or response times was conducted.

**Table 1.** Cross-theme synthesis of implementation barriers, enablers, and practical implications.

Theme	Main barriers	Main enablers	Practical implication	Transferability
Clinical relevance and initial buy-in	Perceived added workload; device reliability concerns; uneven voluntary engagement	Perceived usefulness for early detection; growing confidence through direct use	Frame telemonitoring as clinical support, not added surveillance; clarify participation expectations	Broadly transferable
Training and onboarding	Variable digital literacy; overlap between training formats; delayed refresher needs	Hands-on device interaction; just-in-time support; local super-user champions	Pair technical onboarding with foundational digital-skills support	Broadly transferable
Workflow integration and alert management	Alert fatigue; fragmented platforms; connectivity issues in rural areas; coverage of unfamiliar patients	Daily/near-daily dashboard access by most nurses; local workflow adaptation; centralized alert triage	Integrate dashboards into routine clinical systems; redesign alert logic and prioritization	Broadly transferable; integration constraints partly context-specific
Professional role tensions and sustainability	No reduction in visit frequency; vendor dependence; lack of interoperability with core systems	Earlier intervention on decompensation; clinical judgment supported by remote data	Treat telemonitoring as augmentation, not replacement; plan governance and interoperability early	Transferable principles; Quebec governance specifics are context-bound

### Clinical Relevance and Initial Buy-In

Nurses held mixed views on the feasibility of telemonitoring. Some were enthusiastic, seeing it as aligned with evolving home care practices and useful for earlier detection and targeted interventions.

“I thought it was really interesting to remotely monitor heart failure patients. Their follow-up is closely tied to vital signs, weight, and symptoms.”

[Nurse #20]

Others voiced reservations about added workload, the reliability of patient-device interaction, and their own comfort with digital tools.

“I was afraid that having to examine all the user data would create extra work… That concern was shared by many nurses.”

[Nurse #15]

In some cases, participation was perceived as encouraged institutionally but not entirely voluntary. These initial attitudes shifted over time, as many nurses reported that direct use of the system increased their confidence and perceived clinical value.

### Training and Onboarding

Nurses received both clinical and technical training prior to deployment, including a self-paced module and a hands-on session delivered by the technology partners. While this format was broadly appreciated, particularly the chance to interact directly with devices, many nurses found overlap between formats. Hands-on exposure was consistently seen as more effective than theoretical content.

In one organization, a supplemental virtual session on the xPill adherence system was well received and helped clarify a tool initially viewed as complex. Training aligned with early deployment allowed just-in-time troubleshooting, while delays led some nurses to forget key elements, highlighting the need for refresher resources.

Managers emphasized that beyond training format or timing, the deeper issue was limited digital readiness and cultural resistance. Many nurses struggled with basic tools like email or Teams, underscoring the need for foundational digital support alongside technical onboarding.

“I think we tend to vastly overestimate digital literacy. Our professionals are really not there yet, even managing basic tools like email or Teams is a challenge. We're still at the foundational level. And when you add new devices on top of that… For me, effective change management has to include education to ensure these basic skills are in place.”

[Manager #1]

### Workflow Integration and Alert Management

Nurses described varied routines for checking the Virtuose dashboard, depending on their workload, comfort with technology, and local workflows. In some sites, alerts were handled by a designated colleague; in others, each nurse monitored their own patients. This difference shaped how the system fit into daily practice. In centralized models, some nurses—especially those with only one enrolled patient—relied on others to manage alerts. While some found the system easy to use, others described it as just one more platform to manage alongside existing tools. They faced challenges moving between systems, repeating information, and trying to reconcile data that did not always match. For some, this added complexity led to overload and reduced confidence in their ability to track patient information accurately.

Alert management was a major operational challenge. Nurses received alerts for clinical thresholds (eg, weight, oxygen saturation), non-adherence, and connectivity issues. Clinical alerts were generally seen as more useful, but the high volume—especially for minor deviations or unfamiliar patients—often caused fatigue and disengagement.

"It became overwhelming when I was covering for colleagues and getting alerts for patients I didn’t know. I just did the bare minimum."

[Nurse #7]

Over time, troubleshooting was increasingly delegated to users or vendor support. Still, unresolved connectivity issues in rural areas often led to unnecessary home visits.

"One recurring issue was poor cellular reception at a patient’s home. I often had to go there just to restart the watch or tablet. These extra visits weren’t clinically useful, just for managing devices."

[Nurse #10]

### Professional Role Tensions and Sustainability

Nurses broadly agreed that telemonitoring enhanced their ability to detect subtle clinical changes and intervene earlier when necessary.

"There were some good interventions, one patient was decompensating and we prevented it from getting worse with Lasix after seeing symptoms in the daily report."

[Nurse #1]

At the same time, many emphasized that raw data alone, particularly when detached from a personal clinical relationship, was insufficient for confident decision-making. Familiarity with the patient remained essential for interpreting alerts.

“One of my patients was desaturating at night. We discovered that. We informed the doctor, but nothing was done. I just left the saturation data there…”

[Nurse #5]

Although the intervention was framed as a potential efficiency tool, few nurses reported any reduction in home visit frequency. Most patients continued to need in-person care for other conditions, and existing visit schedules were already minimal. Several questioned the added value of intensive remote monitoring for clinically stable users.

Concerns about long-term sustainability were also prominent. Participants stressed the need for institutional ownership, clear governance, and integration into core workflows. Many viewed the ongoing reliance on vendors and ad hoc coordination as misaligned with the demands of routine public care delivery.

Professionals adapted pragmatically but pointed to structural limitations that must be addressed for telemonitoring to scale in public home care.

## Discussion

Overall, implementation was shaped by perceived clinical usefulness, hands-on support, alert burden, and limited system integration. This analysis contributes to the digital health implementation literature by centering on frontline providers rather than patient outcomes [28], highlighting professional adaptation, system-level constraints, and sustainability tensions in public home care.

### Implementation Enablers and Barriers

Several factors supported the successful onboarding of nursing staff. Training that combined self-paced video modules with in-person, hands-on sessions was generally effective, especially when timed with early patient deployments. Nurses valued real-time support during installations, often finding it more useful than theoretical preparation. This aligns with research emphasizing the importance of active facilitation and practical guidance in digital health adoption [29].

Engagement with the Virtuose dashboard varied, with some nurses deferring to centralized monitoring owing to low patient load or digital discomfort. Frustrations included redundant alerts, poor prioritization, and notification about unfamiliar patients, consistent with alert fatigue in clinical settings [30].

Technical challenges also limited integration. Frequent device disconnections, connectivity issues, and user adherence problems sometimes required in-person troubleshooting, undermining the model’s efficiency. Although nurses eventually redirected technical problems to vendor support, these disruptions eroded confidence in the system’s reliability, consistent with reported unintended consequences of telehealth deployment [31,32].

### Clinical Integration and Professional Adaptation

Telemonitoring was generally perceived as clinically useful, particularly for detecting weight fluctuations, respiratory symptoms, or oxygen desaturation associated with heart failure decompensation. Nurses appreciated the ability to intervene earlier and more selectively, especially for patients at higher risk. However, participants underscored that data alone was not sufficient for confident decision-making. When alerts involved unfamiliar patients or when values fell within chronic variability rather than true deterioration, nurses hesitated to act without contextual insight. In that sense, the technology served as a decision support tool rather than a decision-making system.

The intervention did not consistently reduce nurse home visits, as many patients required in-person care for other conditions and baseline schedules were already minimal. Nurses questioned the value of telemonitoring for clinically stable users, suggesting a need for clearer patient-selection criteria—findings consistent with other implementation studies [33].

### System-Level Challenges and Sustainability

Beyond workflow integration, the study revealed structural and governance-level issues affecting long-term sustainability. Nurses expressed concern about the system’s dependence on external vendors for technical support and updates, and the lack of integration between the telemonitoring dashboard and their primary clinical software, which fragmented workflows and increased cognitive load. These concerns reflect broader challenges in digital health sustainability, particularly in systems reliant on proprietary platforms without long-term procurement and governance strategies [34].

Budget planning and financial oversight were managed by the MSSS, which funded the project as part of a pilot initiative over an 18-month period. The budget covered device acquisition, software licensing, training, technical support, and coordination activities. Although detailed financial breakdowns were not shared with the research team, implementation partners indicated that vendor contracts and licensing fees represented a substantial portion of total costs. Equipment was acquired via direct contracts between each CISSS and the technology partners, and biomedical engineering departments in each CISSS handled device identification, calibration, maintenance, and end-of-pilot retrieval, reset, and secure storage for potential reuse.

The pilot was designed as an evidence-to-policy exercise rather than a vendor-led commercialization: findings and recommendations were submitted to the MSSS to inform a potential managed expansion across the Quebec network, with decisions on continuation, broader deployment, or hand-over remaining with the ministry at the time of writing.

Interoperability limitations further constrained implementation. The dashboard’s lack of integration with core clinical systems forced staff to navigate additional platforms, while the xPill adherence system required community pharmacists to subscribe to a proprietary interface, limiting participation and undermining the utility of adherence alerts. These challenges underscore the importance of interoperable design, alignment with existing infrastructure, and coordinated sector-wide implementation [34].

Taken together, several implementation lessons are broadly transferable to other public or community-based home care settings: the importance of hands-on training for variable digital readiness, alert-fatigue mitigation, and the continued centrality of relational clinical judgment. However, certain challenges—reliance on proprietary platforms without interoperability standards, pilot-based MSSS funding, and CISSS governance structures—are specific to Quebec’s public system. Successful scale-up elsewhere would require interoperable infrastructure, sustained institutional ownership beyond pilot phase, and alignment with local nursing workflows and capacity.

### Trustworthiness

Trustworthiness was supported through inclusion of diverse perspectives across sites and roles, use of consistent interview guides, systematic and iterative coding, collaborative theme development, and grounding interpretations in participant quotations. Detailed contextual description supports transferability, and team-based analysis and reflexivity strengthened confirmability.

### Limitations

Despite the rigor of our approach, this study has certain limitations. First, it does not present cross-site comparisons. While this was a deliberate design choice, consistent with the collaborative and staggered nature of the implementation, and with the homogeneity of experiences reported across sites, it limits the ability to assess region-specific variations in implementation. Second, the analysis centers primarily on the experiences of home care nurses, who were the primary users of the telemonitoring tools. Although managerial and technical perspectives were included for context, this focus may exclude broader organizational dynamics. Third, the study captures early implementation experiences and does not evaluate long-term impacts on clinical outcomes or sustained integration into routine practice. Finally, given the qualitative design and limited number of participating regions and professionals, the findings are not statistically representative and should be interpreted as context-specific insights rather than generalizable conclusions.

### Recommendations

The following conditions appear essential for health systems seeking to replicate this implementation (Table 2).

**Table 2.** Practical recommendations for successful telemonitoring implementation.

Condition for success	Practical recommendation
Institutional alignment	Secure commitment across clinical, managerial, and technical levels before deployment; define roles and responsibilities explicitly
Workflow integration	Embed dashboards into existing clinical systems; avoid standalone platforms that increase cognitive load
Realistic efficiency expectations	Frame telemonitoring as complementary, not substitutive; ensure adequate staffing and time
Interoperability and external partners	Plan shared platforms and equitable access for all actors (eg, community pharmacists) from the outset
Implementation prerequisites	Protect implementation leadership; train for clinical interpretation and technical use; predefine alert ownership; select patients carefully; plan vendor support beyond installation; assess interoperability early

## Supplementary material

10.2196/83615 Multimedia Appendix 1 Illustrative examples of quotes, codes, and themes showing the coding-to-theme process.

10.2196/83615 Checklist 1 i-CHECK-DH (Guidelines and Checklist for the Reporting on Digital Health Implementations) checklist

## Abbreviations

CISSS: Health and Social Services Centers (Centres intégrés de santé et de services sociaux)
HF: heart failure
MSSS: Ministry of Health and Social Services (Ministère de la Santé et des Services sociaux in French)
