# Supplementary material for: Implementation of a Multidevice Telemonitoring Program for Home-Based Nursing Care in Quebec: Qualitative Report
Source: JMIR Med Inform. 2026 Jun 16;14:e83615. doi: 10.2196/83615 (PMC13271516; doi:10.2196/83615)
Supplement: Multimedia Appendix 1 [file medinform-v14-e83615-s001.docx]

**Multimedia Appendix 1. Illustrative examples of quotes, codes, and themes showing the coding-to-theme process**

| **Theme** | **Code** | **Exemplar Quote** | **Source** |
| --- | --- | --- | --- |
| Clinical Relevance and Initial Buy-In | Perceived clinical utility | *“I thought it was really interesting to remotely monitor heart failure patients. Their follow-up is closely tied to vital signs, weight, and symptoms.”* | Nurse #20 |
| Clinical Relevance and Initial Buy-In | Anticipated workload burden | *“I was afraid that having to examine all the user data would create extra work… That concern was shared by many nurses.”* | Nurse #15 |
| Training and Onboarding | Digital literacy as prerequisite | *“I think we tend to vastly overestimate digital literacy. Our professionals are really not there yet, even managing basic tools like email or Teams is a challenge. We’re still at the foundational level. And when you add new devices on top of that… For me, effective change management has to include education to ensure these basic skills are in place.”* | Manager #1 |
| Training and Onboarding | Gap between technical and clinical training | *“There was no training on how to interpret the data in the console, on what to do with that data… We were focused on how to use the technology, without the clinical reasoning behind it.”* | Manager #2 |
| Workflow Integration and Alert Management | Alert fatigue under coverage conditions | *“It became overwhelming when I was covering for colleagues and getting alerts for patients I didn’t know. I just did the bare minimum.”* | Nurse #7 |
| Workflow Integration and Alert Management | Connectivity-driven unnecessary visits | *“One recurring issue was poor cellular reception at a patient’s home. I often had to go there just to restart the watch or tablet. These extra visits weren’t clinically useful, just for managing devices.”* | Nurse #10 |
| Professional Role Tensions and Sustainability | Clinical value of early detection | *“There were some good interventions, one patient was decompensating and we prevented it from getting worse with Lasix after seeing symptoms in the daily report.”* | Nurse #1 |
| Professional Role Tensions and Sustainability | Relational care limits data-only decisions | *“One of my patients was desaturating at night. We discovered that. We informed the doctor, but nothing was done. I just left the saturation data there…”* | Nurse #5 |
| Professional Role Tensions and Sustainability | Technology as organizational shift | *“We must do things differently. We need to introduce tools that lead people to adapt how they work.”* | Manager #3 |

*All interviews were conducted in French; quotes have been translated by the first author. Participant codes replace names throughout.*
