# Supplementary material for: Implementation of a Multidevice Telemonitoring Program for Home-Based Nursing Care in Quebec: Qualitative Report
Source: JMIR Med Inform. 2026 Jun 16;14:e83615. doi: 10.2196/83615 (PMC13271516; doi:10.2196/83615)
Supplement: Checklist 1 [file medinform-v14-e83615-s002.docx]

**i-CHECK-DH checklist**

*Manuscript: “Implementation of a Multidevice Telemonitoring Program for Home-Based Nursing Care in Quebec: A Qualitative Report.” Mandatory items are flagged (M); non-mandatory items are flagged (NM). Locations refer to section headings in the revised manuscript.*

**Legend: Reported** = item clearly addressed in the manuscript. **Partially reported** = item addressed but with one or more sub-elements missing. **Not reported** = item missing and recommended for revision. **Not applicable** = item not relevant to this implementation, with justification.

| **i-CHECK-DH item** | **Reported / Partially reported / Not reported / Not applicable** | **Location in manuscript** | **Supporting text or brief justification** |
| --- | --- | --- | --- |
| **Title** | | | |
| **1. Title (M)** | **Reported** | Section header and title | Manuscript opens with the section header “Implementation Report” and the title “Implementation of a Multidevice Telemonitoring Program for Home-Based Nursing Care in Quebec: A Qualitative Report,” explicitly identifying the article as an implementation report and naming the intervention. |
| **Abstract** | | | |
| **2. Abstract (M)** | **Reported** | Abstract (Background, Objective, Methods, Results, Conclusions) | Structured abstract follows the recommended order. It summarizes the implementation context (Quebec, three CISSS, MSSS-funded pilot), the intervention (four connected devices and the Virtuose dashboard), the qualitative methods, the implementation findings, and the conclusions. No formal KPIs are reported because the article is qualitative; this is consistent with item 6 and is stated explicitly in the Methods. |
| **Introduction** | | | |
| **3. Context (M)** | **Reported** | Introduction; Methods – Study Context | Geographical area (Quebec), implementing organizations (three CISSS), target population (older adults with heart failure receiving home care), and rural/urban diversity are described. The pilot is explicitly placed under the mandate of the Quebec MSSS Direction générale des aînés et des proches aidants and framed as evidence generation for potential province-wide scale-up; in iCHECK-DH terms, the implementation is identified as being at the Piloting and Evidence Generation stage. |
| **4. Problem statement (M)** | **Reported** | Introduction (paragraphs 1–3) | The healthcare problem is clearly stated: aging populations, workforce shortages, rising chronic disease burden, and the specific clinical and organizational challenges of heart failure management in home care. The gap addressed (limited real-world evidence on telemonitoring implementation in public home care) is articulated. No reference to the WHO Classification of Digital Health Interventions “health system challenge” is included; this is optional under iCHECK-DH but could be added. |
| **5. Similar Interventions (M)** | **Reported** | Introduction | The manuscript states that prior Quebec studies have evaluated home telemonitoring for chronic obstructive pulmonary disease and hypertension, and that to the authors’ knowledge no implementation of multidevice telemonitoring for heart failure had previously been conducted within Quebec’s public home care system. The added value of the present pilot is articulated as the combination of four connected devices, a centralized nurse-facing dashboard, and integration into routine CISSS home-care workflows in three regions. |
| **Methods** | | | |
| **6. Aims and Objectives (M)** | **Reported** | Methods – Study Aim and Design; Abstract – Objective | Aims (feasibility, acceptability, integration into nursing practice; identification of barriers, enablers, and contextual adaptation strategies) are clearly stated. The manuscript explicitly explains why no formal KPIs are reported: the article focuses on the qualitative, lived experience of implementation, and quantitative components are not in scope. iCHECK-DH allows this provided a detailed explanation is given, which is the case. |
| **7. Blueprint summary (M)** | **Reported** | Methods – Study Context; Intervention and Nurse Involvement | The intervention design and key features are described: four connected devices (Bluetooth scale, smartwatch, xPill adherence system, voice-activated tablet), centralized Virtuose dashboard, daily symptom questionnaire, nurse-led monitoring, multi-site governance, staggered but unified deployment with shared protocols and training materials. |
| **8. Technical Design (M)** | **Reported** | Methods – Intervention and Nurse Involvement | Architecture is described (proprietary, closed-source platforms by Virtuose Technologies and DOmedic; Virtuose cloud hosting; threshold-based automated alerts; pharmacist access via DOmedic interface) and licensing status is stated (proprietary, vendor-owned). The rationale for tool choice is now explicit: a co-development partnership formalized at project inception, with the founder of Virtuose Technologies as a project co-instigator alongside the academic lead, and equipment acquired via direct contracts between each CISSS and the technology partners under the ministerial research mandate, without a public tender. Regulatory status at acquisition is reported (smartwatch Health Canada-approved; connected scale not). Sub-items such as open-source documentation and project-website links are not applicable given the closed-source vendor model. |
| **9. Target (M)** | **Reported** | Methods – Study Context; Participants and Data Collection | Implementation sites (three CISSS in Quebec, rural and urban) and patient eligibility (French-speaking adults aged 65+, receiving home care for heart failure, no neurocognitive disorder) are specified. Nurse target population (21 home care nurses) and managerial participants are described. |
| **10. Data (M)** | **Reported** | Methods – Intervention and Nurse Involvement; Ethical Considerations | Privacy impact assessment and cybersecurity testing prior to deployment are reported. Data hosting (Virtuose cloud), patient consent procedures, encrypted storage of research data, de-identification of transcripts, and compliance with provincial data protection laws are described. External access to patient data (community pharmacists) is conditioned on explicit patient consent. Granular data lifecycle elements (e.g., post-pilot data retention, deletion) and final IP ownership of clinical data are not exhaustively detailed but the core governance picture is reported. |
| **11. Interoperability (M)** | **Reported** | Methods – Intervention and Nurse Involvement; Discussion – System-Level Challenges | The manuscript explicitly states that the Virtuose dashboard operated independently from existing clinical systems (i-CLSC, SyMO) with no FHIR-based interoperability, and that the xPill adherence system required a proprietary pharmacist interface. The absence of standards is reported as a finding rather than masked, which satisfies the item. |
| **12. Participating entities (M)** | **Reported** | Methods – Study Context, Intervention and Nurse Involvement; Discussion | Implementing organizations (three CISSS, Virtuose Technologies, DOmedic) are named, and government involvement (Quebec MSSS as funder and selector of pilot sites) is described. The vendor-owned status of the platforms is noted, addressing IP ownership. Roles of partners (training, technical support) are described in the Intervention section. |
| **13. Budget Planning (M)** | **Partially reported** | Discussion – System-Level Challenges and Sustainability | The funding source (MSSS), the period covered (18 months), and the budget categories (devices, software licensing, training, technical support, coordination) are reported. The contractual mode is now stated (direct contracts between each CISSS and the technology partners), and the equipment lifecycle is described (biomedical engineering departments handled identification, calibration, maintenance, and end-of-pilot retrieval, reset, and secure storage for potential reuse). Specific dollar figures and a percentage breakdown are not disclosed; the manuscript explicitly notes that detailed financial breakdowns were not shared with the research team and that vendor contracts and licensing fees represented a substantial portion of total costs. Item remains Partially reported because no figures or ranges are publicly available. |
| **14. Sustainability (M)** | **Reported** | Results – Professional Role Tensions and Sustainability; Discussion – System-Level Challenges; Recommendations (Table 2) | Sustainability tensions (vendor dependence, lack of interoperability, MSSS pilot funding, ad-hoc governance, prerequisites for institutionalization) are discussed at length, and Table 2 lists conditions for successful replication. The exit/institutionalization strategy is now explicit: the pilot was designed as an evidence-to-policy exercise rather than a vendor-led commercialization, with findings and recommendations submitted to the MSSS to inform a potential managed expansion across the Quebec network; decisions on continuation, broader deployment, or hand-over remain with the ministry at the time of writing. |
| **Implementation (Results)** | | | |
| **15. Coverage (M)** | **Reported** | Results (opening paragraph) | Coverage is sub-national / regional: three CISSS in Quebec. Numbers reported: 67 patients initially recruited, 34 completed the 6-month intervention; 19 nurses and 11 managers/coordinators interviewed. Relative coverage as a percentage of the eligible Quebec home-care heart-failure population is not reported, which is appropriate given the pilot scope. |
| **16. Outcomes (M)** | **Reported** | Results (themes 1–4); Table 1 | Implementation outcomes are reported as four qualitative themes (clinical relevance and initial buy-in; training and onboarding; workflow integration and alert management; professional role tensions and sustainability), summarized in Table 1, with one process indicator (~76% of nurses accessed the dashboard daily or near-daily). Health outcomes are not reported; the manuscript states this is out of scope and that no formal KPIs were tracked, consistent with item 6. |
| **17. Lessons learned (M)** | **Reported** | Discussion (all subsections); Recommendations – Table 2 | Success factors (hands-on training, just-in-time support, local super-users, perceived clinical usefulness), challenges (alert fatigue, fragmented platforms, connectivity issues, vendor dependence, limited interoperability), and budget-related observations are discussed. Recommendations for replication are consolidated in Table 2 (institutional alignment, workflow integration, realistic efficiency expectations, interoperability, implementation prerequisites). Transferability is addressed in Table 1 and in the closing paragraph of the Discussion. |
| **18. Unintended consequences (NM)** | **Reported** | Results – Workflow Integration and Alert Management; Discussion – Implementation Enablers and Barriers | Unintended consequences are explicitly discussed: alert fatigue and disengagement; additional in-person home visits to restart devices in areas with poor cellular reception; cognitive load from navigating multiple non-integrated platforms. The manuscript situates these findings within the broader telehealth unintended-consequences literature. |
| **Discussion** | | | |
| **19. Conclusion (M)** | **Reported** | Abstract – Conclusions; closing paragraphs of Discussion | Conclusions summarize that telemonitoring can be implemented in public home care given strong leadership, responsive training, and adaptive workflows, and identify the relational, technical, and organizational dimensions to address. Future implications for scale-up in similar public health systems are stated, including the requirement for interoperable infrastructure and sustained institutional ownership. |
| **General** | | | |
| **20. General (NM)** | **Reported** | Methods – Ethical Considerations; Acknowledgements; Funding; Conflicts of Interest | Ethics approval is reported (REB MP-23-2023-1037, July 11, 2023; institutional agreements at the two additional sites). Conflicts of interest are declared (AC associate editor of JMIR). Generative AI use during manuscript preparation is disclosed in Acknowledgements per JMIR Publications guidelines. The Funding section appropriately distinguishes between the MSSS-sponsored pilot (disclosed in Introduction and Discussion) and the absence of author-specific funding for the qualitative analysis and manuscript preparation, which is a routine and acceptable distinction in implementation reports. Trial/study registration is not mentioned; given the qualitative implementation design and existing REB approval, registration is not required. |
